# Prediction of acute myeloid leukemia prognosis based on autophagy features and characterization of its immune microenvironment

**DOI:** 10.3389/fimmu.2024.1489171

**Published:** 2024-11-22

**Authors:** Chaoqun Zhu, Xiangyan Feng, Lanxin Tong, Peizheng Mu, Fei Wang, Wei Quan, Yucui Dong, Xiao Zhu

**Affiliations:** ^1^ School of Computer and Control Engineering, Yantai University, Yantai, Shandong, China; ^2^ Department of Hematology, Yantai Yuhuangding Hospital Affiliated to Qingdao University, Yantai, Shandong, China; ^3^ Guangzhou Dublin International College of Life Sciences and Technology, South China Agricultural University, Guangzhou, Guangdong, China; ^4^ Department of Immunology, Binzhou Medical University, Yantai, Shandong, China

**Keywords:** autophagy gene, immune infiltration, random forest, acute myeloid leukemia, prognosis

## Abstract

**Background:**

Autophagy promotes the survival of acute myeloid leukemia (AML) cells by removing damaged organelles and proteins and protecting them from stress-induced apoptosis. Although many studies have identified candidate autophagy genes associated with AML prognosis, there are still great challenges in predicting the survival prognosis of AML patients. Therefore, it is necessary to identify more novel autophagy gene markers to improve the prognosis of AML by utilizing information at the molecular level.

**Methods:**

In this study, the Random Forest, SVM and XGBoost algorithms were utilized to identify autophagy genes linked to prognosis, respectively. Subsequently, six autophagy genes (TSC2, CALCOCO2, BAG3, UBQLN4, ULK1 and DAPK1) that were significantly associated with patients’ overall survival (OS) were identified using Lasso-Cox regression analysis. A prediction model incorporating these autophagy genes was then developed. In addition, the immunological microenvironment analysis of autophagy genes was performed in this study.

**Results:**

The experimental results showed that the predictive model had good predictive ability. After adjusting for clinicopathologic parameters, this feature proved an independent prognostic predictor and was validated in an external AML sample set. Analysis of differentially expressed genes in patients in the high-risk and low-risk groups showed that these genes were enriched in immune-related pathways such as humoral immune response, T cell differentiation in thymus and lymphocyte differentiation. Then immune infiltration analysis of autophagy genes in patients showed that the cellular abundance of T cells CD4+ memory activated, NK cells activated and T cells CD4+ in the high-risk group was significantly lower than that in the low-risk group.

**Conclusion:**

This study systematically analyzed autophagy-related genes (ARGs) and developed prognostic predictors related to OS for patients with AML, thus more accurately assessing the prognosis of AML patients. This not only helps to improve the prognostic assessment and therapeutic outcome of patients, but may also provide new help for future research and clinical applications.

## Introduction

Acute myeloid leukemia (AML) is a complex and diverse blood cancer triggered by abnormal proliferation and immature differentiation of hematopoietic stem cells in the bone marrow (1–3). Despite previous studies on the role of autophagy in AML (4–6), the specific functions of ARGs and their interaction with immune infiltration have not been thoroughly explored. This gap not only makes the biological functions of ARGs unclear, but also limits their potential application in AML therapy. Therefore, this study aimed to reveal the key autophagy genes associated with the prognosis of AML and their role in relation to the immune microenvironment through comprehensive bioinformatics analysis, providing new targets and strategies for AML treatment.

Autophagy is an important cellular self-regulatory mechanism that maintains cellular and organismal homeostasis (7) and adapts to changes in the external environment by disassembling and removing damaged proteins and organelles inside the cell. Autophagy gene (ARG) mutations linked to cancer and other diseases (8). For example, autophagy levels are strongly associated with the prognosis of ovarian cancer patients (9). Recent studies have indicated that autophagy is closely linked to progression of leukemia, including AML. However, the exact mechanism of autophagy in AML and the expression and function of autophagy genes in AML are still limited.

Certain immune cells play an immunoregulatory role in the tumor microenvironment (TME) and are linked to the immune escape of tumor cells, thereby influencing tumor progression (10). Bansal et al. showed that the number of regulatory T cells was significantly higher in patients with AML than in the healthy population, and that the increased number of Tregs may be strongly associated with poor prognosis (11). Wan et al. further noted that Tregs contribute to immune escape of AML cells in the tumor microenvironment by enhancing the inhibitory effect on effector T cells (12). The study by Romee et al. demonstrates the potential of using cytokines to induce memory-like NK cells for immunotherapy in AML patients (13). Bioinformatics analysis of immune infiltration is a powerful approach that allows in-depth study of immune cell infiltration in TME and its relationship with tumor development by integrating multi-omics data. Although there have been several studies on immune infiltration in AML, the interaction between ARGs and immune infiltration has not been thoroughly investigated.

**Figure 1:**
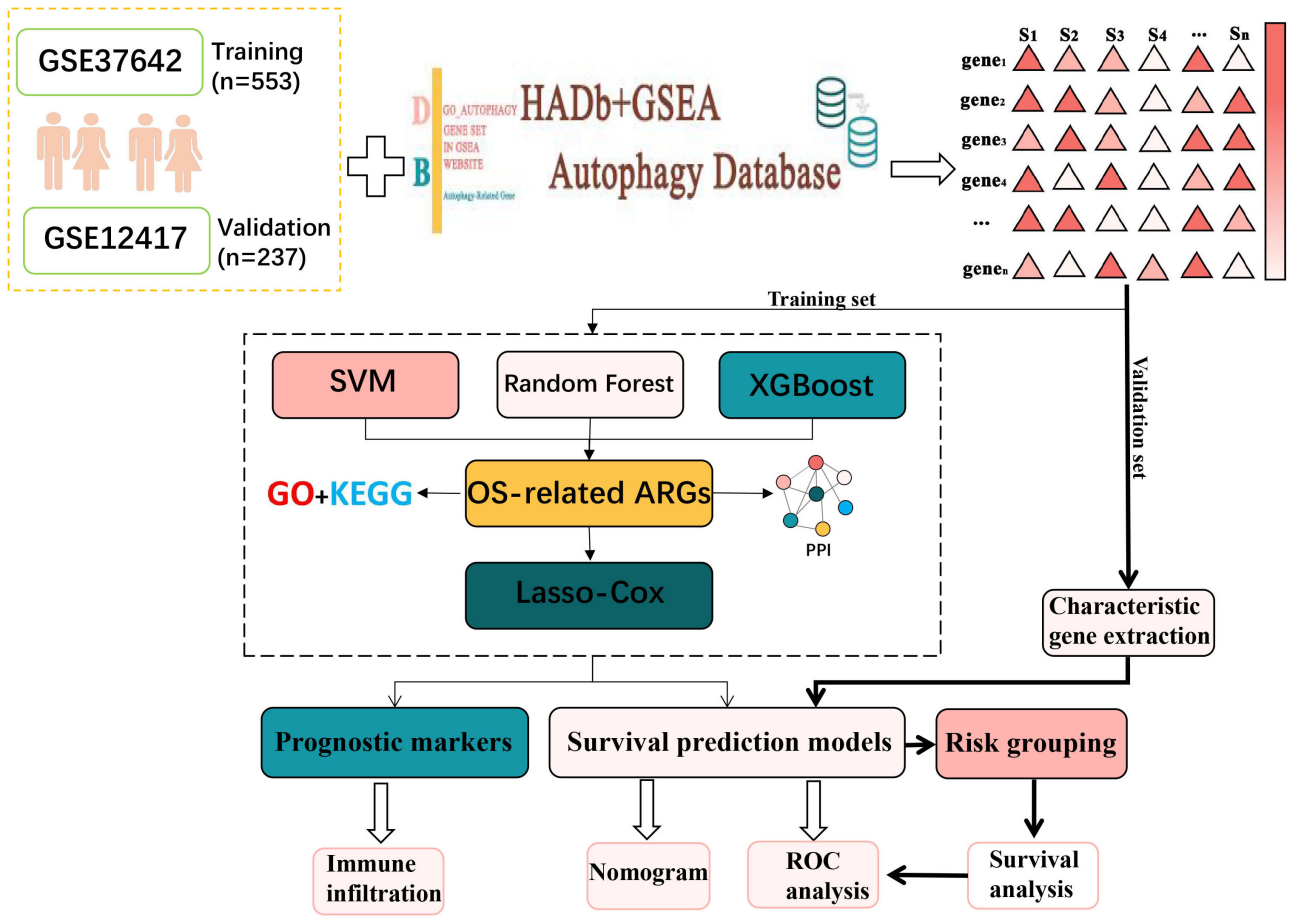
The general analytical flow of this experimental design.

In this research, AML transcriptome data obtained from the GEO database was used to screen for AML-related ARGs (14–16). Then functional enrichment analyses were conducted to obtain the biological meaning and functional features of these ARGs. In addition, the autophagy genes obtained in this experiment were analyzed by protein–protein interaction (PPI) network to obtain the interactions between these autophagy genes and their regulatory mechanisms inside the cell. After that, Random Forest (17), SVM-RFE (18) and XGBoost were used in combination to identify key autophagy genes associated with AML. Lasso-Cox analysis was then conducted to identify six autophagy-related genes and construct a survival prediction model. After that, AML high and low risk groups divided according to the survival prediction model and differential expression analysis was performed. The genes obtained with significant differences were then analyzed for functional enrichment. The results indicated that these ARGs were primarily enriched in immune-related pathways such as T cell differentiation in thymus and lymphocyte differentiation. Accordingly, the autophagy genes were analyzed for immune infiltration. Moreover, the link between ARGs and immune infiltration was investigated. This study reveals the critical role of autophagy genes in acute myeloid leukemia and their interaction with the immune microenvironment, which is of great clinical significance. By constructing a survival prediction model, it can provide AML patients with prognostic assessment and personalized treatment plans. In addition, autophagy genes are expected to be used as potential targets for novel therapeutic strategies, especially showing great potential in combination with immunotherapy. The basic flow of this experiment is shown in **Figure 1**.

## Methods

### Data set acquisition

In this study, the original microarray dataset of GSE37642 (19) was downloaded from the GEO database, including transcriptome data of GPL96 and GPL570 platforms. The data were first quality checked for missing values, outliers and distribution. Subsequently, the data were normalized using the robust multi-array average (RMA) algorithm in the affy package to normalize gene expression levels across arrays. To eliminate the batch effect due to different platforms, the Combat algorithm from the sva package was used for correction (20). Clinical information was then collated and integrated to remove samples lacking relevant clinical information, resulting in 553 usable acute myeloid leukemia samples. Clinical information on these samples is presented in **Supplementary Table S1**. The dataset GSE12417 was processed in the same way, resulting in a total of 237 samples with clinical information. The available clinical information for the samples used was shown in **Supplementary Table S2**. AML RNA-seq datasets were downloaded from the UCSC Xena database (https://xenabrowser.net/datapages/). Available clinical information for the samples used in this study is shown in **Supplementary Table S3**.

### Acquisition of autophagy genes

Autophagy-related genes were obtained from the Human Autophagy Database (HADB, http://www.autophagy.lu/index.html) and from the GO_AUTOPHAGY gene set in GSEA website (http://software.broadinstitute.org/gsea/index.jsp). The Human Autophagy Database (HADb) is an authoritative database dedicated to autophagy-related genes, covering a large number of experimentally validated autophagy genes, which ensures the breadth and comprehensiveness of the data. The collection of GO_AUTOPHAGY genes on the GSEA website is based on the autophagy biological process as defined by Gene Ontology (GO), and these genes are strictly classified according to the GO classification criteria division, ensuring consistency in biological function and annotation. This enables the autophagy genes selected during the study to have a clear functional orientation and ensures their relevance to the autophagy process. The two obtained autophagy gene sets were combined to obtain 531 related ARGs (**Supplementary Table S4**). 392 ARGs were screened from GSE37642.

### Random forest identifies overall survival-related ARGs

In this study, survival time and survival state information were extracted from AML patient data, and a random forest model with 1000 decision trees was constructed to predict patient survival. The model used multiple samples, each containing feature genes and their corresponding survival information. To build the decision trees, the random forest employed the log-rank split rule, which assessed the survival differences between two subsets. At each candidate split point, the log-rank statistic was calculated to measure the difference between two survival curves, using the formula $X^{2}=\sum_{i=0}^{m}\frac{\left(O_{i}-E_{i}\right)}{E_{i}}$ , where ​ $O_{i}$ was the observed number of events at time point i ,​ $E_{i}$ was the expected number of events at i , and m was the total number of time points. The split point with the highest log-rank statistic is selected as the optimal point, as it maximizes the distinction between the survival curves of the resulting subsets. This process continues recursively, splitting the data at the best split points until a stopping condition is met.

### SVM identifies ARGs

In this study, a SVM model was used to identify the most important features for the classification task. The importance of each feature was determined by looking at how much influence it had on the model’s decisions. This was done by calculating the absolute value of the product of the feature’s weight and the corresponding support vector. In simpler terms, the importance of a feature depends on how much its weight, when multiplied by the support vector, affects the classification. After calculating these importance scores, they were sorted from highest to lowest to identify the most important features.

### XGBoost Identifies ARGs

Firstly, the training data were preprocessed, including extracting the autophagy gene expression data from the transcriptome data, and also collecting clinical information such as the survival time and survival status of the patients. Handle missing and abnormal values to ensure complete autophagy gene expression data for each sample and remove abnormal or incomplete samples. Generate labels by combining survival time and survival status. Next, the data are converted to DMatrix format for XGBoost and the model parameters are set, where the objective function is Cox proportional risk model and the evaluation metric is negative log likelihood. The objective function of Cox proportional risk model (21) is defined as:


(1) $$logL\left(\beta \right)=\sum_{i\in E}\left(x_{i}\beta -\text{log}\left(\sum_{j\in R\left(T_{i}\right)}\text{exp}\left(x_{j}\beta \right)\right)\right)$$

where E denotes the set of events, i.e., all samples of observed deaths, $R\left(T_{i}\right)$ denotes the set of samples at risk at time $T_{i}$ . $x_{i}$ dentes the eigenvector of sample i , and β is a parameter of the model. The model is trained through 100 rounds of iterations, setting the learning rate to 0.1, and recording the negative log-likelihood value and training error for each round as a function of the number of iterations. The model is then used to calculate the importance of the features. Feature importance (22) (Gain) indicates the contribution of each feature to the model with the following formula:


(2) $$Gain\left(j\right)=\sum_{t\epsilon T_{j}}\text{Δ}G_{t}$$

where $\Delta G_{t}$ denotes the gain of feature j in tree t and $T_{j}$ denotes the set of all trees in which feature j  appears.

### Permutation test

To further assess the impact of the identified ARGs on survival, a permutation test was conducted. This test aims to verify the reliability of the model’s predictions by randomly shuffling the survival labels, as described below.

1. Randomly disrupt survival state labels to generate a new set of labels.
2. Retrain the Cox regression model using the disrupted data and record the C-index of the model each time.
3. Repeat the above process a certain number of times to generate a C-index replacement distribution.
4. The C-index of the original model was compared with the C-index distribution of the replacement and the p-value was calculated to assess the significance of the original model.

### Functional enrichment analysis and PPI molecular interactions

Gene ontology (GO) and Kyoto Encyclopedia of Genes and Genomes (KEGG) analyses of key autophagy genes were performed using clusterProfiler (version 3.14.3) to reveal the primary functions of these genes. We will apply the Benjamini & Hochberg correction method and use a corrected P value of less than 0.05 as the criterion for statistical significance.

To study the interactions between these key ARGs, a PPI will be constructed using the STRING database. Subsequently, the MCODE plugin was used in Cytoscape (v3.10.0) (23) to extract densely connected modules with default parameters “degree cutoff = 2”, “node score cutoff = 0.2”, “K-core = 2”, and “Maximum depth = 100” to extract densely connected modules.

### Construction and validation of survival prediction models

To avoid overfitting of prognostic risk features, we performed the following steps on the training set to construct survival prediction models.

1. A Cox regression method based on the least absolute shrinkage and selection operator (LASSO) was applied to the training dataset to identify significant features of ARGs associated with OS.
2. Subsequently, we performed multivariate Cox proportional risk regression on these candidate genes and stepwise variable selection using the Akaike information criterion.
3. Ultimately, risk scores for optimized prognostic markers were calculated.

(3) $$Risk\;score=\sum_{i}^{n}Coef_{i}\times A_{i}$$

where $Coef_{i}$ represents the regression coefficient of the i gene, indicating the degree of influence of the expression level of the gene on the risk. $A_{i}$ denotes the expression level of the i gene, and n denotes the total number of genes selected for characterization. Differences in patient OS were assessed by Kaplan-Meier analysis and log-rank tests. The predictive power of ARG-based characteristics was assessed using time-dependent ROC curves (24).

To test the accuracy of the survival prediction model, external validation was performed using the GSE12417 (*n*=242) dataset and AML cohorts-TCGA-LAML (*n*=129). First, the risk scores of patients in each external validation dataset were calculated using the survival prediction model from the training set. Then, patients were categorized into high-risk and low-risk groups based on their risk scores. Next, the survival distribution of the model in the high- and low-risk groups was assessed using Kaplan-Meier curves, and the survival differences were compared to validate the predictive performance of the model.

### Identification of differentially expressed genes

Differential expression analysis was performed on samples from the high-risk and low-risk groups using the limma package, setting the criteria of |log2FC| > 2 and a P-value < 0.05 to screen for DEGs. Next, volcano maps of DEGs were plotted using the EnhancedVolcano (25) function in the EnhancedVolcano package.

### Immune infiltration analysis

The analysis of 22 immune cell types is of great importance during the progression of AML. These immune cells, including T cells, B cells, NK cells, T cells gamma delta and macrophages, are known to play a key immunomodulatory role in the tumor microenvironment (26). Ge Jiang et al. demonstrated that a significant elevation in the abundance of NK cells and macrophage infiltration was strongly associated with a poor prognosis in AML (27). Another study by Moore et al. demonstrated that macrophage reduction promoted AML cell growth *in vivo* (28).

To further investigate the relationship between immune cell infiltration and AML, the CIBERSORT algorithm was used to calculate the infiltration abundance of 22 immune cell types in gene expression data from AML patients. Subsequently, the association between hub genes and the abundance of 22 immune cells was detected and then visualized using the software package “ggcorrplot”, and gene-immune cell correlations greater than 0.28 were considered significant.

## Results

### Using machine learning to select OS-related ARGs

Three hundred and ninety-two ARGs were screened from the gene expression matrix and screened for autophagy genes associated with survival prognosis using Random Forest, Support Vector Machine (SVM) and XGBoost (29) algorithms, respectively.

**Figure 2:**
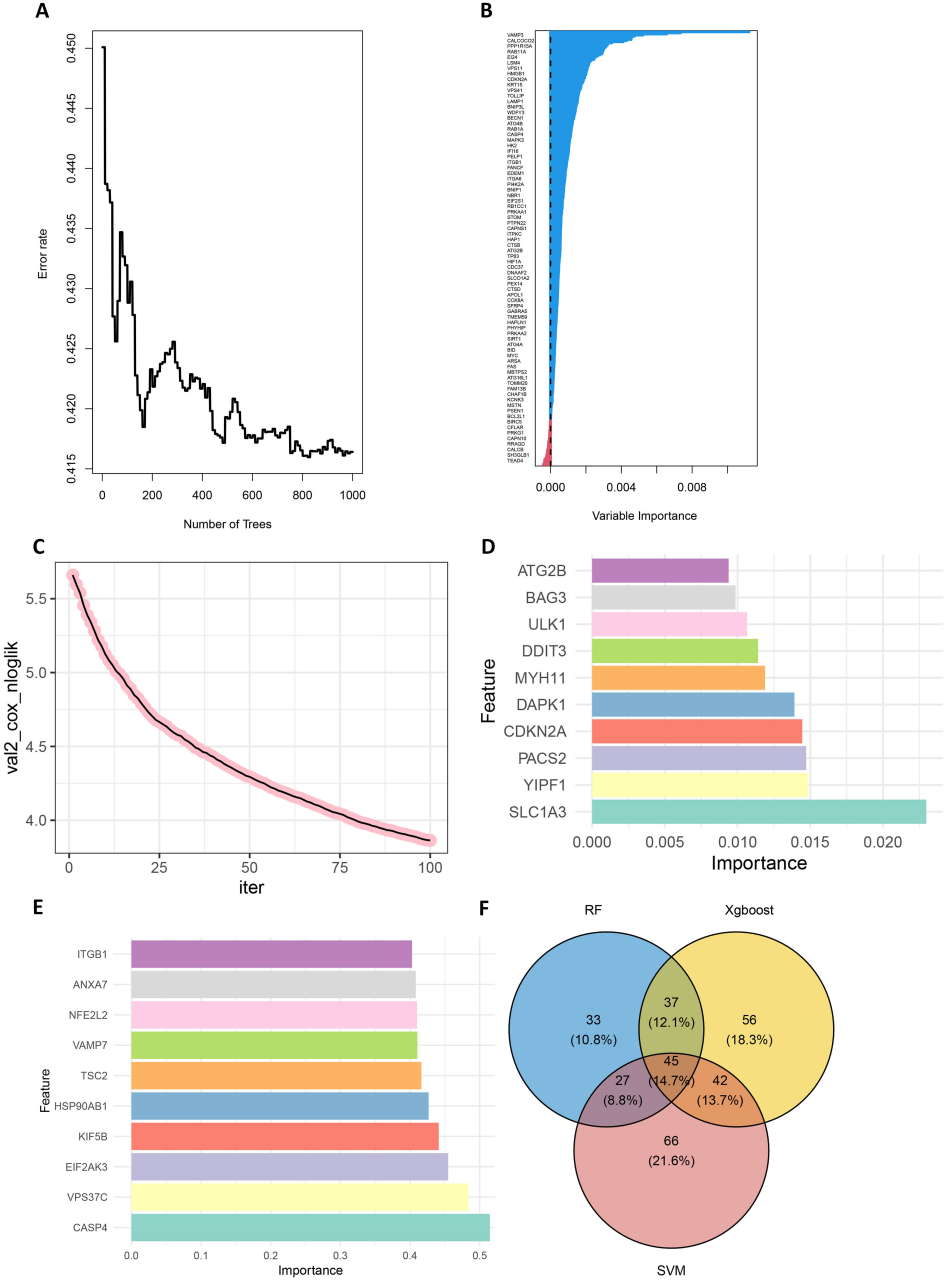
Screening for prognostically relevant autophagy genes using machine learning methods. **(A)** The OBB error plot of the random forest algorithm is used to estimate the generalization performance of the model. The graph shows that as the number of trees increases, the errors of model become smaller. **(B)** VIMP plot showing the importance scores of each variable to help identify the most important feature genes. **(C)** Plot of the number of iterations of the training process of the XGBoost algorithm versus the Cox negative log-likelihood value. **(D)** Bar chart of the top 10 genes and their corresponding importance scores screened by the XGBoost algorithm **(E)** Bar chart of the top 10 genes and their corresponding importance scores screened by the SVM algorithm. **(F)** Venn plots of overlapping genes shared by SVM, Random Forest and XGBoost.

First, in the random forest model, 1000 decision trees were constructed and the variables were partitioned using the log-rank rule. The model assessed the relationship between gene expression and survival prognosis by calculating the importance of each variable and the proximity of the samples (30). The OBB error plot of the model showed a gradual decrease in error and improved performance as the number of trees increased (**Figure 2A**). The variable importance plot showed the importance of each gene (**Figure 2B**), and 146 genes with significant effects on survival analysis were screened (**Supplementary Table S5**). Meanwhile, the performance of the model was assessed by the C-index (consistency index), and a C-index value of 0.88 was obtained, indicating that the model was predicted relatively well.

Next, the XGBoost algorithm was employed for survival analysis. XGBoost used the Cox proportional risk model as the objective function and evaluated the model by optimizing the Cox negative log-likelihood ratio (cox-nloglik). Survival states and survival times were converted into a labelled format suitable for the Cox model, and the number of iterations of the model was set to 100 with a learning rate of 0.1. **Figure 2C** shows the trend of Cox negative log-likelihood value during the training process. From the figure, it can be seen that the model gradually converges and the performance of the model gradually improves as the number of iterations increases. The top 180 genes that had a significant effect on survival analysis were screened by feature importance analysis (**Supplementary Table S6**) and the performance of the model was assessed with a C-index of 0.99. The top 10 ranked important features are shown in **Table 1**. **Figure 2D** visualizes the top 10 ranked genes and their corresponding importance scores. These features had the highest importance scores in the model and significantly influenced survival prediction.

**Table 1:** The top 10 important genes of XGBoost and their importance scores.

ID	Feature	Gain
1	SLC1A3	0.022978235
2	YIPF1	0.014842549
3	PACS2	0.014716427
4	CDKN2A	0.014447571
5	DAPK1	0.013909943
6	MYH11	0.011887605
7	DDIT3	0.011406339
8	ULK1	0.010654985
9	BAG3	0.00985662
10	ATG2B	0.009385788

In addition, a support vector machine (SVM) was used for survival analysis, and a linear kernel function (31) and epsilon regression type were used for model training. The coefficients and support vectors of the model were used to calculate the importance scores of each feature, and the top 180 feature genes that had a significant effect on survival prediction were filtered out (**Supplementary Table S7**), and the top 10 features with the highest importance scores were visualized by bar graphs to show the importance scores of these feature genes (**Figure 2E**). The model has a C-index of 0.17. **Table 2** demonstrates the top 10 significant feature genes and their importance scores. A total of 45 overlapping genes common to all three algorithms were screened by the above algorithm (32) (**Figure 2F**).

**Table 2:** SVM top 10 significant genes and their importance scores.

ID	Feature	Gain
1	CASP4	0.514479265
2	VPS37C	0.48390987
3	EIF2AK3	0.454698135
4	KIF5B	0.441155029
5	HSP90AB1	0.426529294
6	TSC2	0.416280361
7	VAMP7	0.410471634
8	NFE2L2	0.410017974
9	ANXA7	0.40822591
10	ITGB1	0.40279245

By comparing the importance scores of the top 10 genes screened by the three algorithms (**Supplementary Figure 1**), it was found that genes such as ITGB1, ANXA7, and ULK1 scored higher across all algorithms, suggesting a significant association of these genes with survival prognosis in AML. In model performance comparisons, XGBoost showed the best performance, while SVM performed relatively poorly. However, although XGBoost leads in prediction, it is too dependent on parameter tuning in the case of small samples and is prone to overfitting if the parameters are not adjusted properly. SVM, on the other hand, is more suitable for handling high-dimensional data with small samples, and although its overall performance is not as good as that of XGBoost, it has a unique advantage in handling data dimensions.

In order to improve the stability and consistency of the screened genes, we adopted a combination strategy of multiple algorithms. By using SVM, Random Forest and XGBoost algorithms to identify prognostic genes from different angles, we further screened the overlapping genes that showed significance in all three algorithms. Finally, we screened 45 overlapping genes in total (**Figure 2F**).

To further validate the impact of the screened ARGs on the survival prognosis of AML patients, we used the replacement test to assess their statistical significance. The results showed that the C-index of the original model was significantly higher than that of most of the replacement models, and was located at the rightmost end of the replacement distribution (**Supplementary Figure 2**). By comparing the C-index of the original model with that of the replacement models, a p-value of 0.0429 was calculated, indicating that the original model was statistically significant in predicting the survival of AML patients, further confirming the importance of the screened ARGs in survival prediction.

### Enrichment analysis of ARGs

**Figure 3:**
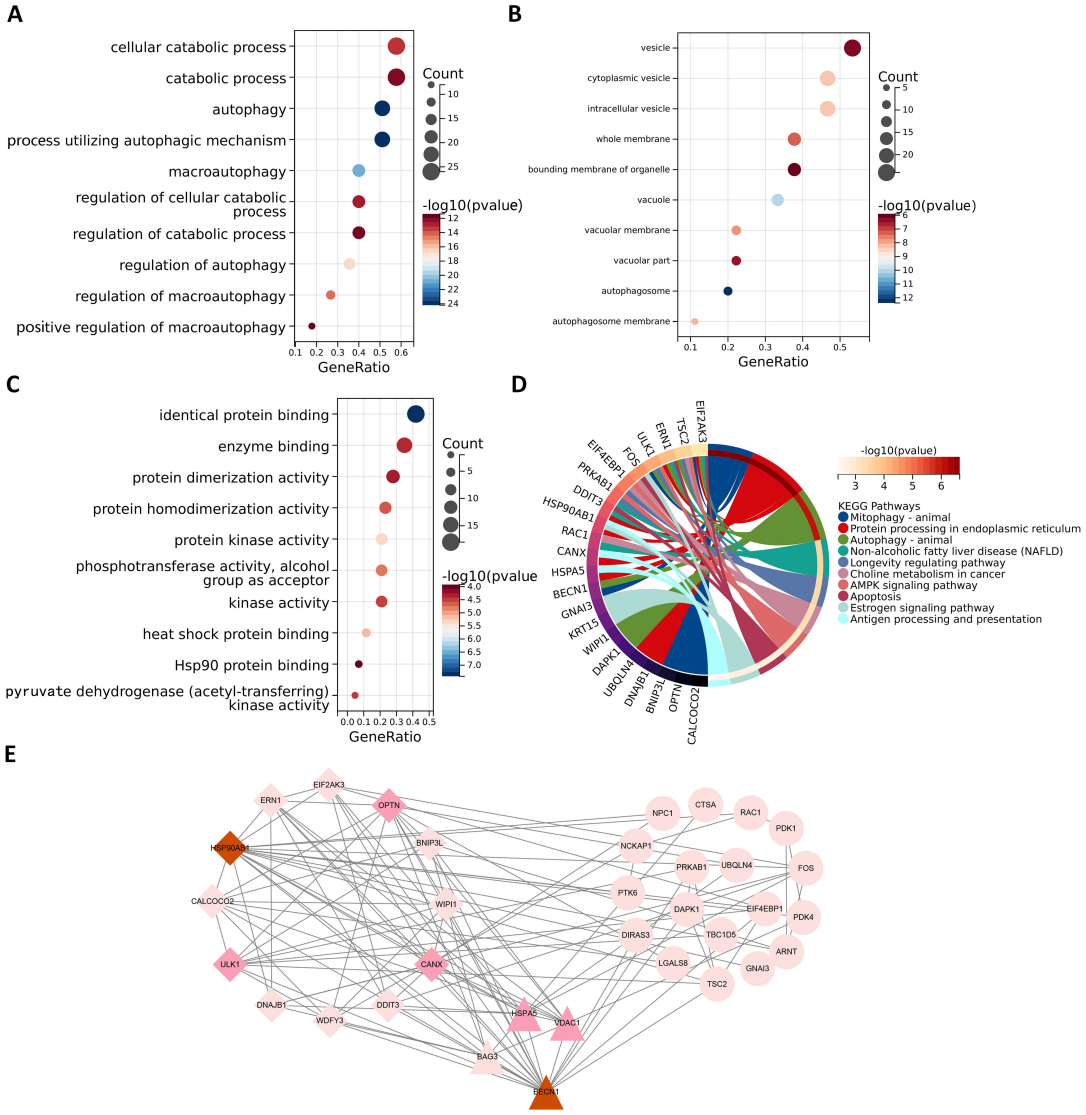
Functional enrichment analysis and PPI analysis of ARGs associated with survival. The gene functional enrichment analysis of key modules **(A)** BP; **(B)** CC; **(C)** MF; **(D)** KEGG pathways. **(E)** HSP90AB1 and BECN1 modules were identified by PPI analysis of ARGs. Darker node colors represent greater node degrees.

In order to better study the biological features in the autophagy gene data so as to understand the functions and regulatory mechanisms of the biological systems, GO and KEGG analyses were conducted. For GO enrichment analysis of autophagy genes, the genes related to total survival were analyzed in terms of biological processes (BP), cellular components (CC), and molecular functions (MF), respectively. BP analysis revealed that these genes were primarily associated with cytolytic metabolic processes, autophagy, and the regulation of processes that utilize autophagic mechanisms (**Figure 3A**). CC analysis indicated that these ARGs were predominantly distributed in cellular components of vesicle, cytoplasmic vesicle and bounding membrane of organelle (**Figure 3B**). MF analysis showed that most of these genes act together on a protein and enzyme with catalytic effects (**Figure 3C**). KEGG revealed that these ARGs were primarily enriched in the pathways of autophagy animal, AMPK signaling and longevity regulation in animals (**Figure 3D**). To gain insight into the interactions between these autophagy genes associated with overall survival, STRING (33) was utilized to construct the PPI network and identify two important modules: the HSP90AB1 module and the BECN1 module (**Figure 3E**). The BECN1 module contains 12 nodes and 29 edges, while the CASP3 module consists of 4 nodes and 6 edges. HSPA5, VDAC1, and BAG3 are the other 3 nodes of the CASP3 module. These ARGs may be important for the pathogenesis of AML.

### Modelling survival predictions

**Figure 4:**
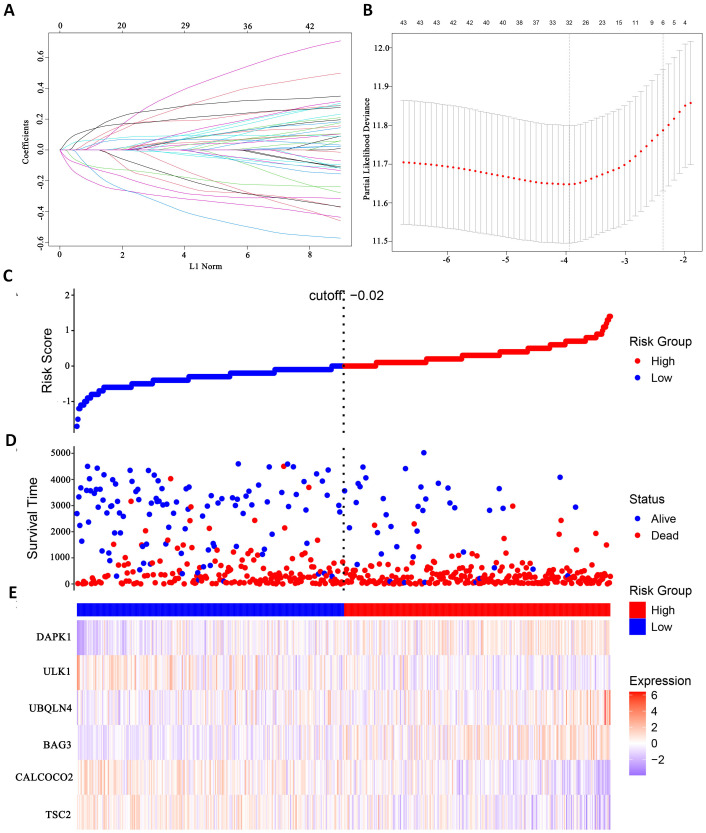
Identify autophagy genes associated with survival. **(A)** Path diagram of LASSO coefficients. **(B)** Cross-validation curve for LASSO regression analysis. **(C)** Change curves of patient risk scores. **(D)** The number of patients corresponding to different survival times. **(E)** Expression of six model genes.

In this study, survival data were systematically analyzed, and feature genes significantly associated with survival were screened by Lasso-Cox regression and used for modelling. First, the optimal lambda value (lambda.1se) of 0.09393562 was selected by 10-fold cross-validation, and the lambda plot and LASSO regression were plotted (**Figures 4A, B**). Next, the non-zero coefficients were extracted and the six characterized autophagy genes and their regression coefficients selected by LASSO were saved. Cox stepwise regression (34) analysis was then conducted to optimize the selection of feature genes (**Table 3**). The resulting risk score model for the patients was as follows:


(4) $$\begin{array}{c} Risk\;score=\left(0.14747\times BAG3\right)-\left(0.14437\times TSC2\right)\\ -\left(0.32652\times CALCOCO2\right)+(0.32410\\ \times UBQLN4)-\left(0.24254\times ULK1\right)+(0.23913\\ \times DAPK1) \end{array}$$

**Table 3:** Survival prediction models for acute myeloid leukemia.

Gene	Coefficients	Exp(coef)	P-value
TSC2	-0.14437	0.86556	0.128973
CALCOCO2	-0.32652	0.72143	0.000299
BAG3	0.14747	1.15890	3.70e-05
UBQLN4	0.32410	1.38278	0.009121
ULK1	-0.24254	0.78463	0.020660
DAPK1	0.23913	1.27015	3.26e-06

Coefficients was the regression coefficient for each variable, indicating the direction and magnitude of the variable's effect on survival time. Se(coef) was the standard error of the regression coefficient for each variable, indicating the uncertainty in the estimation.

Risk scores were subsequently calculated for each sample, and the samples were divided into high and low risk groups based on the median risk score. An increase in the risk score was correlated with a higher number of patient deaths (**Figures 4C, D**). Among the characterized genes screened, DAPK1, UBQLN4, and BAG3 were highly expressed in high risk, and ULK1, ALCOCO2, and TSC2 were highly expressed in low risk (**Figure 4E**).

**Figure 5:**
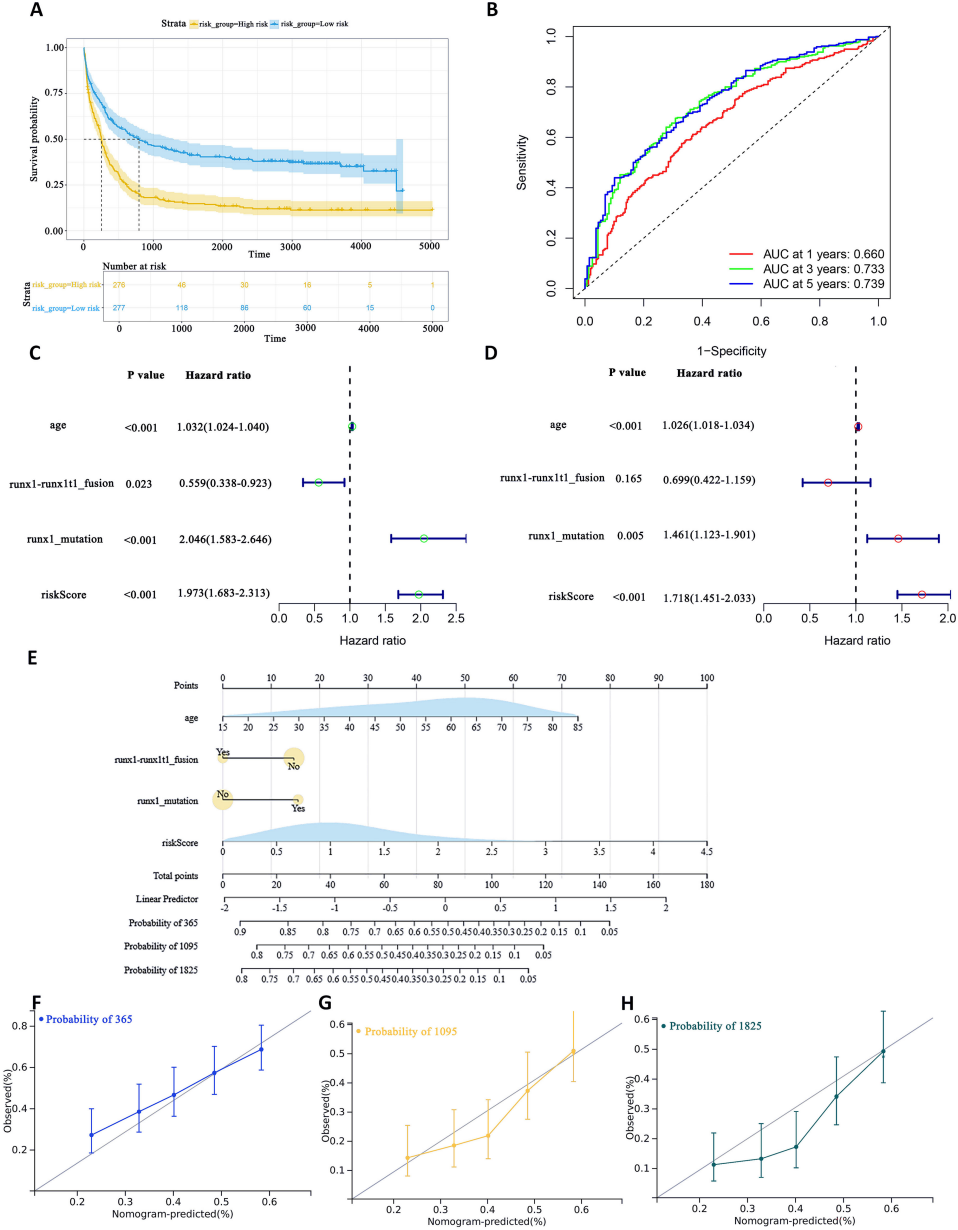
To assess the predictive accuracy of survival prediction models for patient OS. **(A)** Kaplan–Meier curves visualizing the difference in survival time. **(B)** AUC curves for prognostic markers. **(C)** UCRA **(D)** MCRA **(E)** Development of autophagic clinicopathological nomograms for the prediction of OS in AML patients by combining risk scores and clinical information. **(F–H)** Calibration curves-predicting 1-, 3-, and 5-year survival in AML patients. Solid lines indicate ideal performance.

To assess the difference in survival time, the Kaplan–Meier survival curves were used (35). The results showed that patients in the high-risk group had a shorter OS than those in the low-risk group (P< 0.0001, **Figure 5A**). The accuracy of the constructed survival prediction model was evaluated, and the results showed that the AUCs of 1-year, 3-year, and 5-year OS were 0.660, 0.733, and 0.739, respectively (**Figure 5B**), which indicated that the survival prediction model constructed by using the prognostic genes screened in this experiment had high predictive ability.

To quantify the relative importance of the screened autophagy genes in the survival prediction model, we employed a game theory-based SHAP value (SHapley Additive exPlanations) technique. By using the SHAP values calculated by the iml package, we provided a quantitative relative importance score for each gene. Analysis of each gene in the model by SHAP value visually demonstrates the contribution of these genes to the prediction of AML survival (**Supplementary Figure 3**). The average contribution of each gene in the model to the prediction is summarized in **Table 4**. As shown in **Table 4**, these autophagy genes have high contribution values in the model, further supporting their key role in AML survival prognosis.

**Table 4:** Relative importance ranking of autophagy genes based on SHAP values.

ID	Feature	Mean(|SHAP|)
1	BAG3	0.23646
2	TSC2	0.12995
3	UBQLN4	0.04845
4	DAPK1	0.04821
5	ULK1	0.01969
6	CALCOCO2	0.01892

Mean(|SHAP|) denotes the mean of the absolute value of the SHAP value for the gene or trait, i.e., the mean of the gene's contribution to the significance predicted by the model.

Univariate Cox regression analysis (UCRA) and multivariate Cox regression analysis (MCRA) were conducted to validate the independence of prognosis-related autophagy gene survival prediction. UCRA revealed that age, runx1 mutation, and risk score were significantly associated with patients’ OS (**Figure 5C**). MCRA indicated that age and risk score were independent predictors for AML patients, respectively (**Figure 5D**).

To more precisely evaluate the survival prediction model’s effectiveness, nomogram plot integrating risk scores and other survival information was constructed. (**Figure 5E**) The calibration curves demonstrated accurate predictions OS in AML patients (**Figures 5F–H**). This suggests that that integrating our risk score with clinical information can enhance the prediction of OS.

### External validation set validation of survival prediction models

**Figure 6:**
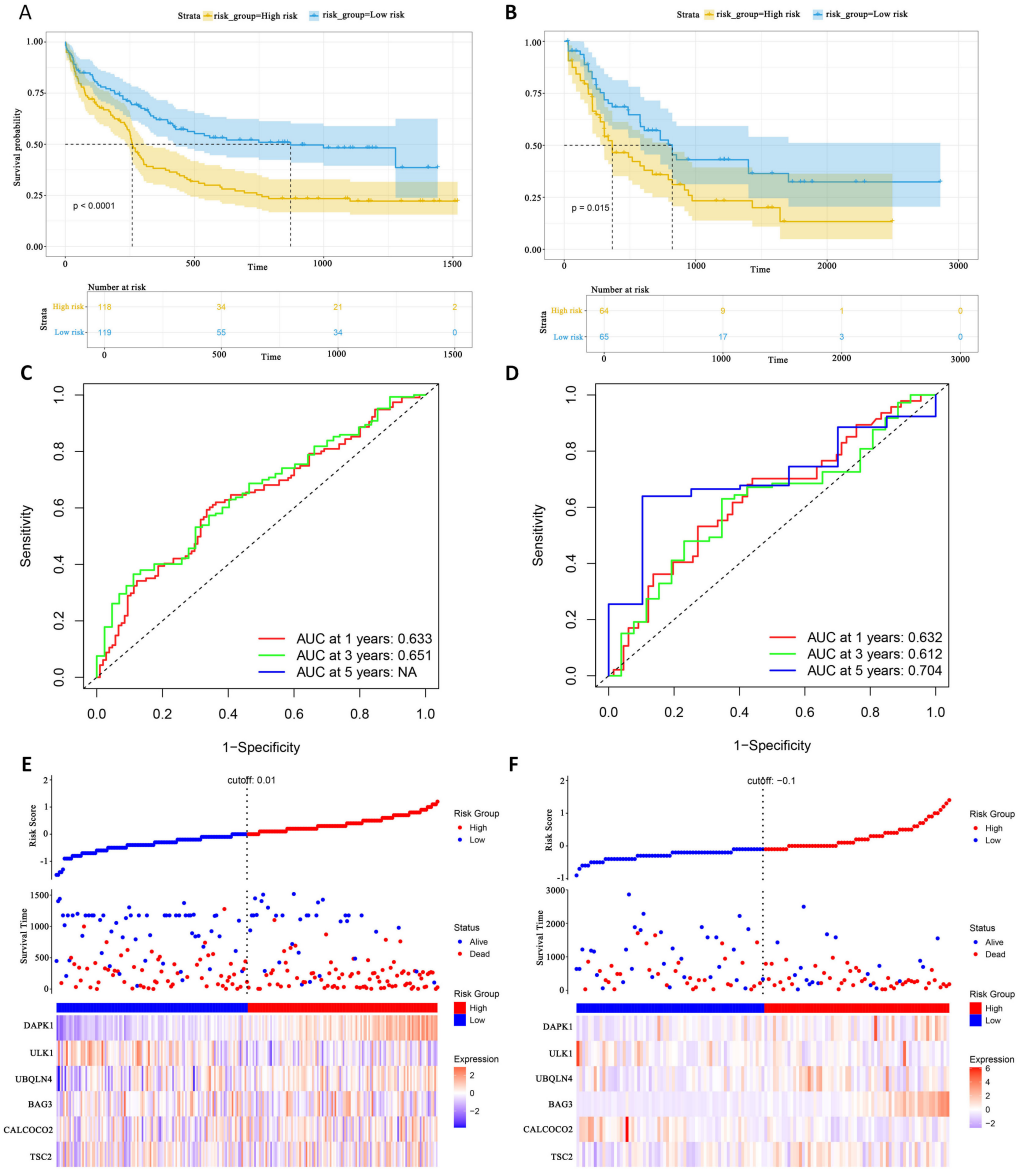
External gene set validation of survival prediction models. **(A, B)** Kaplan-Meier curves of prognostic genes in validation sets GSE12417 and TCGA-LAML. **(C, D)** AUC curve of the GSE12417 validation sets and TCGA-LAML. **(E, F)** Risk score distribution, survival status and 6 prognostic genes expression heatmap in the GSE12417 and TCGA-LAML cohorts.

This study evaluated the diagnostic performance of the models in two external independent validation groups, GSE12417 and TCGA-LAML. Comparison of OS using Kaplan-Meier curves (36) and the log-rank test revealed that in the GSE12417 group, patients in the high-risk group had significantly shorter OS compared to those in the low-risk group (P<0.0001, **Figure 6A**). Similarly, in the TCGA-LAML group, the prognosis of patients in the high-risk group was significantly worse than that in the low-risk group (P=0.015, **Figure 6B**).

To further evaluate the classification performance of the model for patient survival on different datasets, ROC curves for patient survival were plotted based on the model risk score. In the GSE12417 group, the area under the curve (AUC) for 1-year and 3-year OS was 0.633 and 0.651, respectively (**Figure 6C**). In the TCGA-LAML group, the AUC values for 1-, 3- and 5-year OS were 0.632, 0.612 and 0.704, respectively (**Figure 6D**). These results demonstrated the strong predictive power of the model in predicting survival in AML patients. Additionally, this study analyzed the distribution of patients’ risk scores and OS, and found that the mortality rate in the high-risk group was higher than that in the low-risk group. In terms of gene expression, the validation group showed that DAPK1, UBQLN4, and BAG3 were significantly up-regulated in the high-risk group, whereas ULK1, ALCOCO2, and TSC2 were significantly down-regulated in the low-risk group (**Figures 6E, F**), which was consistent with the risk score calculation. Overall, the validation results indicated that the proportional risk model has reasonable accuracy and discriminative ability for independently predicting OS in AML patients.

### Identification and enrichment of DEGs

**Figure 7:**
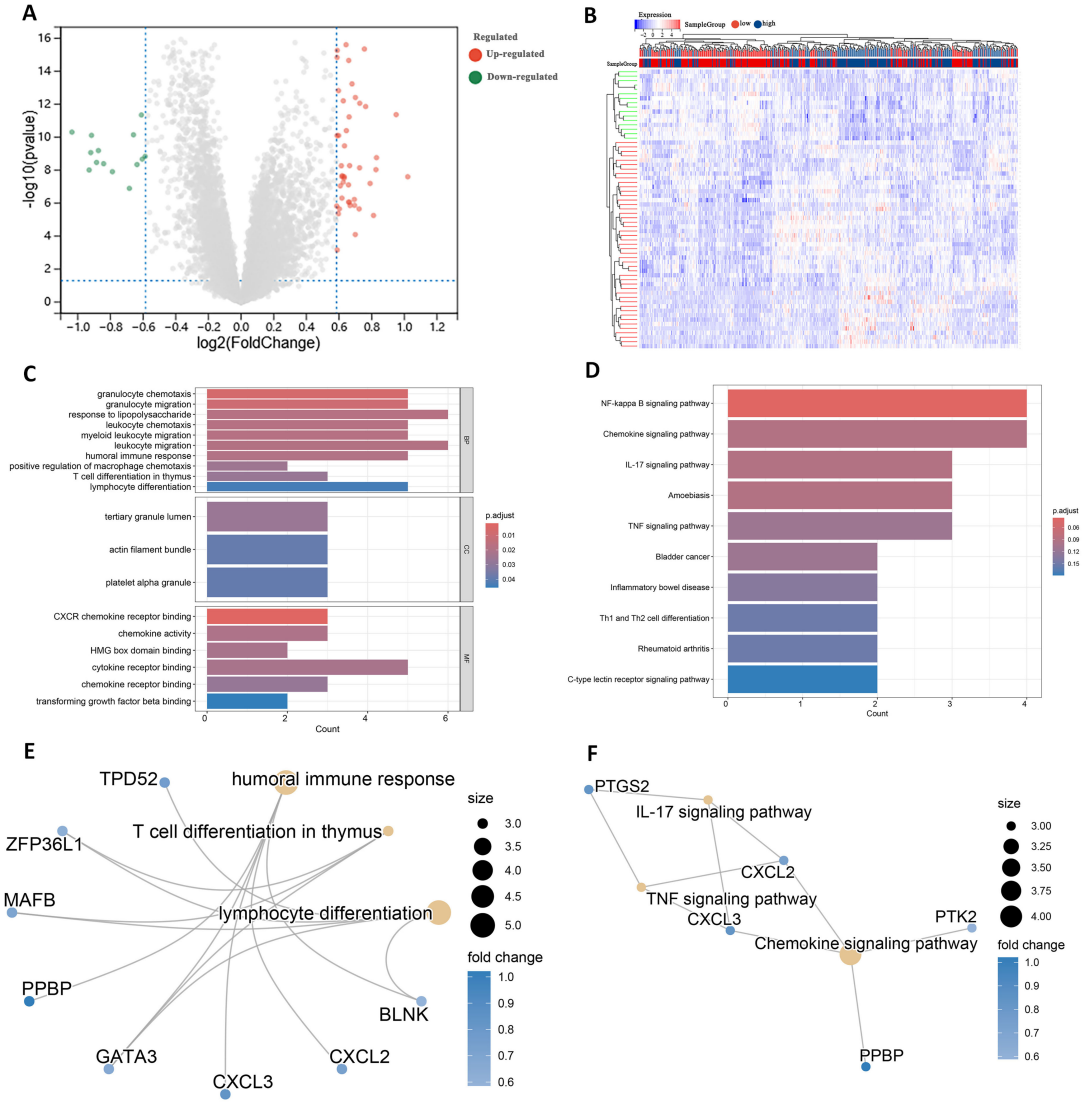
Identification of DEGs in high and low risk groups. **(A)** Volcano plot of DEGs. **(B)** Heatmap of DEGs. **(C)** GO terms analysis of differential genes. **(D)** KEGG pathway enrichment analysis. **(E)** Network diagram of GO terms enrichment with differential genes. **(F)** Network diagram of KEGG pathway enrichment of differential genes.

Differential expression analysis of transcriptome data from patients in the high- and low-risk groups using the limma package identified 63 DEGs, including 47 up-regulated genes and 16 down-regulated genes (**Figure 7A**). The expression patterns of the differential genes are shown in **Figure 7B**. GO enrichment analysis revealed that these DEGs were mainly associated with BP such as T cell differentiation in thymus and lymphocyte differentiation. In terms of cellular components, these genes are predominantly found in the tertiary granule lumen, actin filament bundle, and platelet alpha granule. They are involved in molecular functions such as chemokine activity and cytokine receptor binding (**Figure 7C**).

KEGG pathway analysis indicated that these DEGs were primarily enriched in the IL-17 signaling pathway and Th1 and Th2 cell differentiation (**Figure 7D**). High and low risk group differential genes enriched in lymphocyte differentiation, humoral immune response and T cell differentiation in thymus associated with immune GO terms were TPD52, ZFP36L1 and GATA3 (**Figure 7E**). **Figure 7F** shows DEGs enriched in KEGG pathways such as IL-17 signaling pathway and so on.

In addition to these genes such as BAG3, DAPK1 and GATA3 are enriched in multiple other GO pathways (**Supplementary Figure 4**), and genes such as CXCL2, CXCL3 and CYP1B1 are also present in multiple other KEGG pathways (**Supplementary Figure 5**). This suggests that these genes play important roles in biological processes. In addition, by analyzing the relationships between the enriched pathways, the GO term network relationship map showed significant correlations between chemokine receptors and term such as activity, humoral immune response, and myeloid leukocyte migration (**Supplementary Figure 6**). The KEGG pathway showed that the IL-17 signaling pathway, Chemokine signaling pathway, and TNF signaling pathway also interacted with multiple other pathways (**Supplementary Figure 7**). The enrichment analysis results suggest that these DEGs may play a role in the prognosis and immune response in AML.

### Immune infiltration and immune interactions

**Figure 8:**
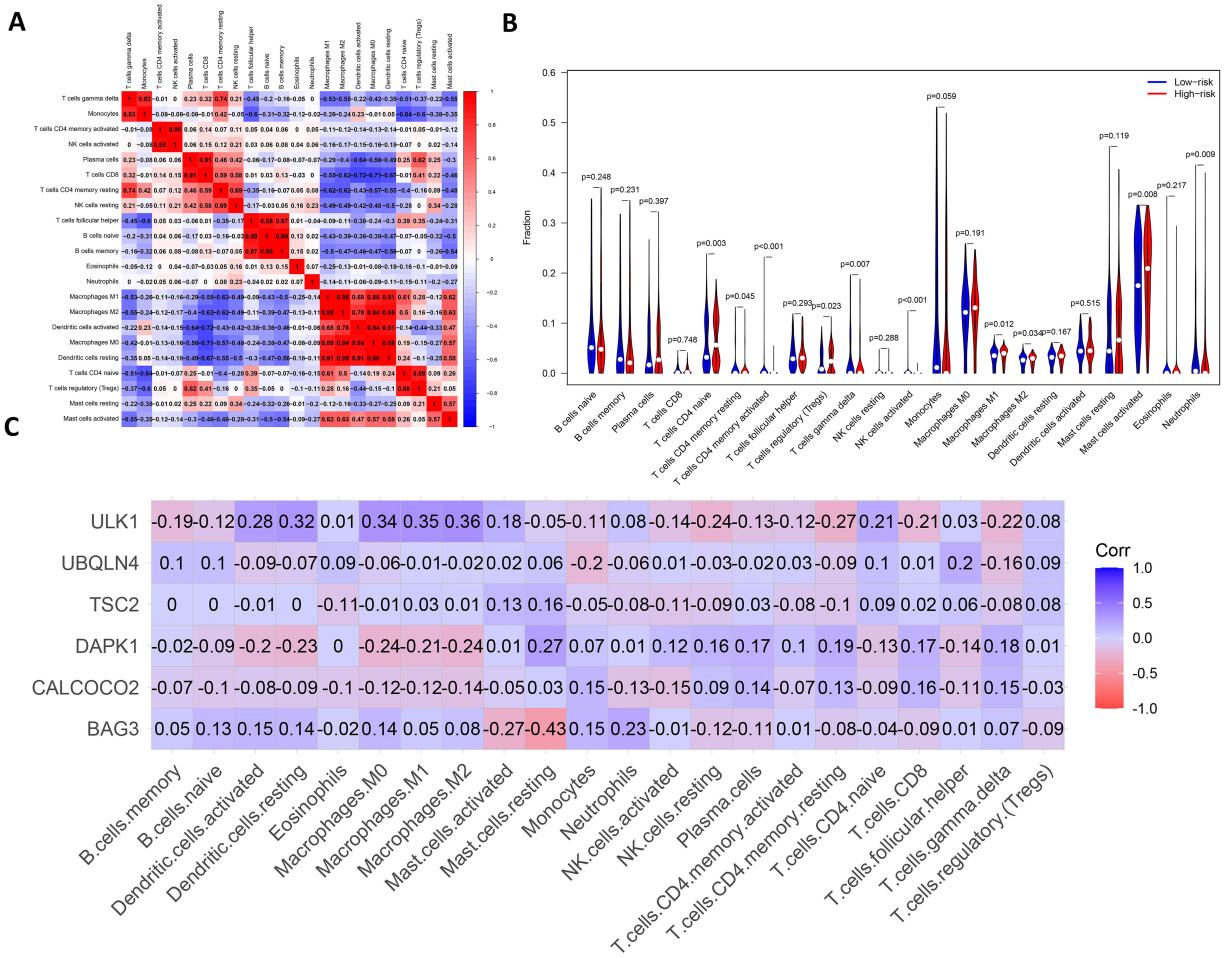
Analysis of leukemia autophagy gene immune infiltration and correlation with hub gene. **(A)** Heatmap of correlation of abundance of different immune cells. **(B)** Violin plots of immune cell abundance. Red represents the high-risk group and dark blue represents the low-risk group. **(C)** Heatmap of the correlation between six prognosis-related genes and immune cell.

There are complex interactions and associations between leukemia and immune infiltration. The immune system was crucial in regulating the development of leukemia. The experiment used the CIBERSORT (37) algorithm to identify 22 subtypes of immune infiltrating cells in AML samples and investigated the interactions of different immune cell subpopulations in AML patients (**Figure 8A**).

**Supplementary Figure 8** shows the ratio of each type of immune cell in AML patients, from which it can be seen that immune cells such as Mast cells activated and Macrophages M0 have a higher ratio in AML. The immune infiltration results indicated that the abundance of immune cells, including T cells CD4^+^ memory activated, NK cells activated and T cells CD4^+^ naive was higher in patients in the low-risk group of AMLs than in the high-risk group (**Figure 8B**). Additionally, the relationship between six key ARGs and immune infiltration was investigated in this experiment. The results showed that these six key ARGs were associated with T cells CD4^+^ naive, T cells CD8^+^, and Macrophages M1, respectively, and immune cells, and changes in the abundance of these immune cells may influence the pathogenesis of AML (**Figure 8C**). The above results suggest that key autophagy genes may affect the abundance of immune cells in AML patients, thereby attenuating the control of leukemia by the immune system and consequently affecting leukemia survival.

## Discussion

Despite significant progress in recent years in the study of prognostic markers for acute myeloid leukemia, the role of autophagy genes in AML is still understudied (38). In this experiment, machine learning methods such as XGBoost, Random Forest and SVM were used to identify potential prognostic markers associated with overall survival in AML (39). Lasso-Cox was then used to further screen for prognostic markers and a survival prediction model consisting of six genes was constructed. The model can predict the overall survival of patients with some generalization ability. In addition, an immune infiltration analysis of autophagy genes in transcriptomic data from AML patients was performed using the CIBERSORT algorithm, and the relationship between identified prognostic markers and immune cell infiltration was analyzed. These analyses have deepened our understanding of TME in AML patients and its impact on disease progression and prognosis.

AML is a severe blood cancer triggered by abnormal proliferation and differentiation of hematopoietic stem cells in the bone marrow. The role of autophagy genes in AML remains under-explored, despite significant progress in the study of AML prognostic markers in recent years. Since AML is a highly heterogeneous disease with multiple molecular features and significant biological differences between patients, it is difficult for a single prognostic marker to accurately predict the prognosis of all patients. Existing studies have mostly focused on common genetic prognostic markers, while studies on autophagy genes are more limited. However, the key role of autophagy in cell survival and death suggests that it may be an important factor influencing AML progression.

The role of autophagy in AML is dual, on the one hand helping AML cells to survive in a hostile environment by removing damaged organelles and proteins (7). On the other hand, autophagy can promote apoptosis in AML cells under certain circumstances (40). In recent years, some studies have begun to explore the role of autophagy in AML. For example, the study by Nan et al. demonstrated that FAT1 inhibited AML cell proliferation by reducing autophagy levels (41), but the study did not delve into the mechanism of the role of specific autophagy genes in AML prognosis. In contrast, Fu et al. used univariate Cox regression to initially screen autophagy genes associated with AML overall survival and further constructed a survival prediction model by Lasso-Cox regression (42). However, univariate Cox regression has limited predictive power when dealing with the complex effects of multivariate on survival. In this study, we conducted a more detailed molecular-level analysis of the relationship between autophagy genes and AML prognosis. We used a screening approach combining three machine learning algorithms, SVM, XGBoost, and Random Forest, which are capable of dealing with complex interactions of multivariate variables and generally provide higher prediction accuracy.

In this study, 45 autophagy genes associated with OS in patients with acute myeloid leukemia were screened using three machine algorithms, SVM, XGBoost and Random Forest algorithm. Among these genes, PDK4 regulates glucose metabolism by inhibiting pyruvate dehydrogenase complex and promotes glycolytic metabolism in tumor cells, an altered metabolism that is a typical feature of cancer cells. Low expression or mutation of BECN1 is closely associated with tumorigenesis. ULK1 plays a crucial role in regulating autophagy in cancer cells. These genes are clinically important as prognostic markers or potential therapeutic targets in cancers such as AML. These autophagy genes were subjected to PPI analysis and then the PPI network was further analyzed using MCODE. As a result, two important modules were identified, namely the HSP90AB1 module and the BECN1 module. It was shown that these ARGs modules have an important impact on OS in AML patients. For example, low expression of BECN1 was associated with poor prognosis in AML patients (43). High expression of ULK1 is associated with better prognosis and it may inhibit tumor growth by promoting autophagy to remove abnormal proteins and damaged organelles from AML cells (4). In addition, genes such as HSP90AB1, CALCOCO2, DNAJB1, and WDFY3 have not yet been extensively studied in the regulation of autophagy in AML. However, these genes are correlated in other cancers (44–46), and they may serve as important prognostic markers in AML. Further pathway enrichment analysis showed that these autophagy genes were mainly enriched in the AMPK signaling pathway, animal autophagy and longevity. It was shown that the activation of AMPK could inhibit the mTOR signaling pathway, promote autophagy and maintain cellular energy homeostasis. By inhibiting lipid and protein synthesis (47), AMPK can limit AML cell proliferation. In terms of GO term these genes are mainly associated with cytolytic metabolic processes, autophagy and the regulation of processes that utilize the autophagic machinery. Decreased cytolytic function is thought to correlate with immunosuppressive status and poor prognosis in AML. For example, Coles et al. showed that upregulation of the immunosuppressive glycoprotein CD200 significantly inhibited the cytolytic capacity of natural killer (NK) cells in AML patients, and that this inhibition reduced the efficiency of the immune system in the clearance of tumor cells, thereby worsening patient prognosis (48). In addition, autophagy, as a key metabolic regulatory mechanism, is closely related to drug resistance in AML cells. a study by Chen et al. indicated that autophagy not only helps leukemia cells to obtain energy and nutrients for metabolism, but also slows down the damage of drugs on AML cells by maintaining intracellular homeostasis under chemotherapeutic stress conditions through metabolic reprogramming (49). Therefore, over-activation of autophagy may make AML cells more resistant to drugs, which in turn affects the prognostic outcome of patients. In summary, cytolysis and autophagy regulation play key roles in the pathogenesis and prognosis of AML, providing a new entry point for the development of future targeted therapeutic strategies.

After Lasso-Cox regression analysis of 45 potential prognostic genes, 6 potential prognostic markers independently affecting AML survival were further screened. Kaplan-Meier analysis showed that the survival rate of the low-risk group was significantly better than that of the high-risk group on both the training. To assess the robustness of the model on different datasets, we validated the constructed survival prediction model using the GSE12417 dataset TCGA-LAML dataset combined with patients’ survival information, respectively. The AUC values for 1-year and 3-year were 0.633 and 0.651, respectively, in the validation set GSE12417.In the validation set TCGA-LAML, the AUC values for 1-year, 3-year, and 5-year OS were 0.632, 0.612, and 0.704, respectively. These results indicate the robustness of the model. Differential expression analysis of patients in the high-risk and low-risk groups showed that these DEGs were mainly enriched in terms such as humoral immune response, T cell differentiation in thymus and lymphocyte differentiation. To investigate the relationship between immune cell abundance and autophagy genes and AML prognosis, an immune infiltration analysis of AML autophagy genes was performed using the CIBERSORT algorithm. The results showed that the abundance of T cells CD4^+^ memory activated, NK cells activated and T cells CD4^+^ naive was higher in patients in the AML low-risk group compared with the high-risk group. This suggests that alterations in the immune microenvironment may make the high-risk group less able to fight cancer. Further investigation of the relationship between these prognostic markers and immune cell abundance showed that ULK1 was positively associated with macrophage subtypes, whereas BAG3 was significantly negatively associated with Mast cells resting, and DAPK1 was negatively associated with multiple immune cell subtypes. DAPK1 was negatively associated with multiple immune cell subtypes. The results suggest that these autophagy genes may regulate AML progression by influencing immune cell infiltration. The underlying mechanisms may involve the central role of autophagy in regulating immune function, with ULK1 promoting anti-tumor immune responses by enhancing macrophage phagocytic activity, BAG3 inhibiting mast cell activity to weaken the immune response, and DAPK1 down-regulation inhibiting the activity of a variety of immune cells, resulting in difficulties for the immune system to recognize and destroy AML cells, which in turn drives tumor progression. In terms of clinical treatment, by targeting the autophagy pathway, it is possible to enhance the activity of specific immune cell subtypes or inhibit the autophagy escape mechanism of cancer cells. For example, activation of ULK1 may enhance the anti-tumor effect of macrophages, whereas by inhibiting BAG3, the control of AML by immune cells may be enhanced. In addition, DAPK1-associated negative regulatory effects could also serve as potential therapeutic targets aimed at restoring the immune system’s ability to recognize and kill AML cells.

In this study, these gene-enriched pathways revealed the critical roles of autophagy and metabolic regulation in the pathogenesis of AML. Autophagy not only helps leukemia cells to meet their metabolic demands, but may also enable AML cells to better adapt to environmental stresses through inter-regulation with, for example, the AMPK signaling pathway. Therefore, targeting these aberrant pathways may provide new strategies for the treatment of AML. Survival prediction models constructed on the basis of these autophagy genes provide more comprehensive and precise prognostic information for personalized treatment of AML patients, helping clinicians to better assess the prognosis of patients and develop personalized treatment plans. In addition, autophagy genes play a key role in regulating immune cell infiltration and its prognostic impact on AML, which provides a research direction to further explore the complex relationship between autophagy and the immune microenvironment. Overall, the study of these pathways is important for an in-depth understanding of the prognostic mechanisms of AML and provides new targets for clinical treatment.

Although this study constructed a prognostic prediction model for AML based on autophagy genes, there are still some limitations. Firstly, although the joint screening of prognosis-related genes by three algorithms, SVM, Random Forest and XGBoost, can combine their respective advantages and improve the stability and consistency of the screening results, XGBoost and Random Forest are susceptible to overfitting when the sample sizes are small, especially when the parameters are not precisely adjusted. In addition, although SVM usually performs better on small sample data, the risk of overfitting may be further amplified when combining these three algorithms. Therefore, special attention needs to be paid to model tuning and validation when applying this combination strategy, especially when dealing with small-sample data, in order to reduce the potential overfitting problem. Secondly, this study mainly relied on transcriptomics data and did not address protein expression or functional status, thus some key biological processes may be missed. Although the model performed well in the validation set, further functional validation and experimental evaluation are needed for its clinical application prospects. Compared with other AML prognostic models, such as Guo et al. (19). who constructed models with common genetic markers or mutation information, our model, although incorporating the specific mechanism of autophagy genes, is slightly deficient in predictive ability, especially the low AUC value in the independent validation set, suggesting that the model’s predictive performance needs to be further improved. In addition, the relatively small sample size of the 2 external validation datasets used in the study may not cover the diversity of AML patients. This limits the ability of the model to generalize to a wider patient population.

Therefore, future studies should further validate the robustness and accuracy of the model in larger and more diverse AML patient cohorts. Meanwhile, in addition to traditional transcriptomics data, multi-omics data such as proteomics and metabolomics can be integrated to provide a more comprehensive biological perspective and avoid missing biological processes that may play a key role in disease development. In addition, functional experiments should be performed on the screened autophagy genes to delve into the specific mechanisms of these genes in AML and to assess their impact on disease progression. In order to gain a deeper understanding of the complexity of the AML tumor immune microenvironment, future studies should be expanded to cover the analysis of more types and subpopulations of immune cells. Finally, based on the importance of these key autophagy genes, precision therapeutic strategies targeting these genes or their associated pathways could be explored in the future, thus promoting further development of personalized treatment for AML patients.

## Conclusion

In this study, we screened six potential autophagy gene prognostic markers for AML (TSC2, CALCOCO2, BAG3, UBQLN4, ULK1, and DAPK1) and constructed a survival prediction model of eight autophagy genes for predicting the survival of AML patients. The model was validated by two validation sets, and the results showed that the survival prediction model had strong validity. In addition, autophagy gene pathway enrichment analysis as well as immune infiltration and immune correlation analysis of ARGs were performed to investigate the biological functions of autophagy genes and the prognostic markers of ARGs in correlation with many immune cells. However, although potential prognostic markers and correlations can be identified from transcriptomic data, the biological significance and clinical application of these results have not been fully confirmed due to the lack of further clinical validation. More medically relevant experiments are needed in the future to validate the potential molecular mechanisms of these genes to better understand their role and application value in AML.

## Data Availability

Publicly available datasets were analyzed in this study. This data can be found here: The public datasets used in this study are the Gene Expression Omnibus (GEO, https://www.ncbi.nlm.nih.gov/, GSE12417 and GSE37642) databases and TCGA databases (https://xenabrowser.net/datapages/).
