# Supplementary material for: Prediction of acute myeloid leukemia prognosis based on autophagy features and characterization of its immune microenvironment
Source: Front Immunol. 2024 Nov 22;15:1489171. doi: 10.3389/fimmu.2024.1489171 (PMC11621098; doi:10.3389/fimmu.2024.1489171)
Supplement: Supplementary file 2 [file DataSheet2.docx]

Supplementary Material

## Supplementary Figures


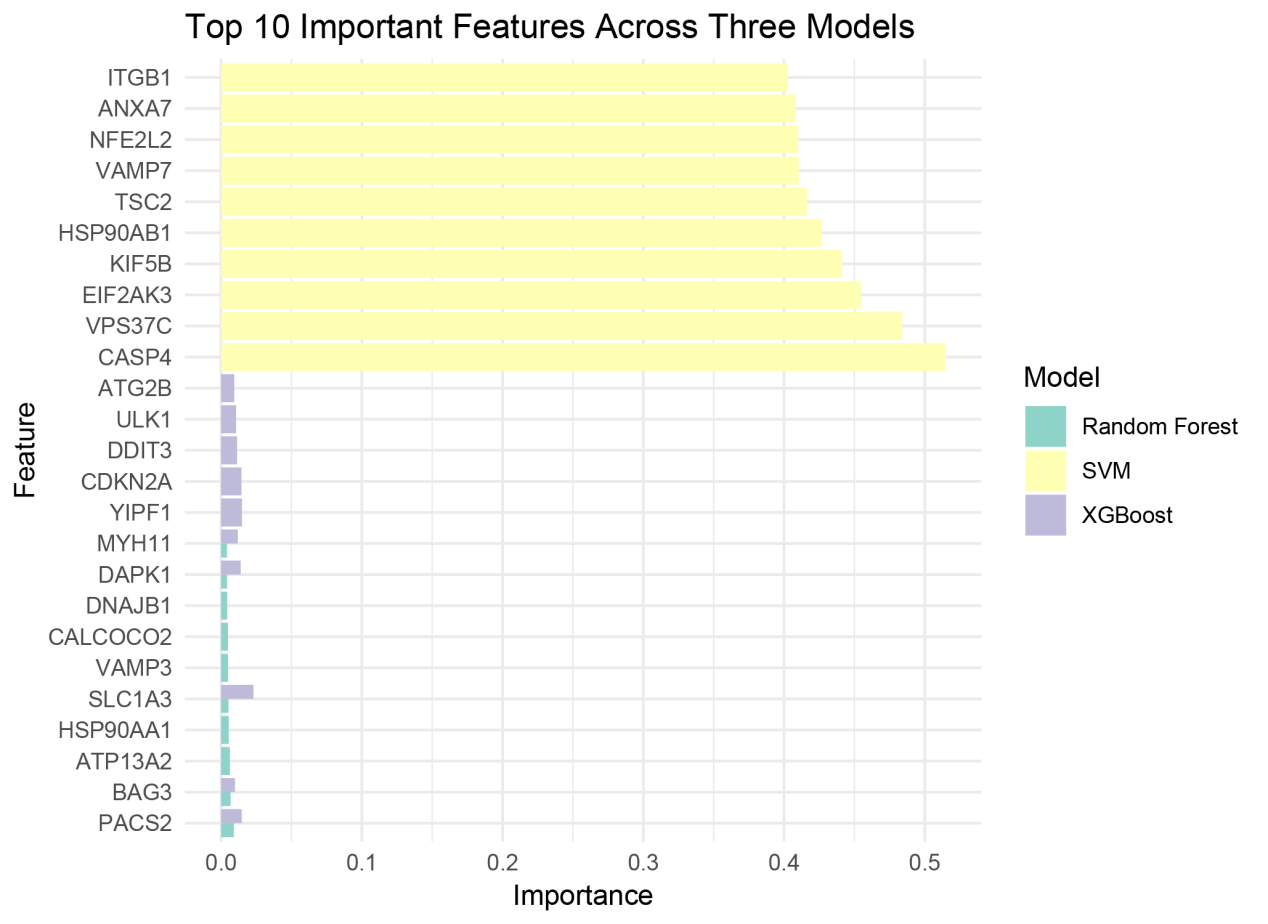


**Supplementary Figure 1.** The top 10 genes of the three-model screen and their importance scores. Rows represent gene names and columns represent the corresponding importance scores. Green represents the importance scores of the top 10 genes screened by the Random Forest algorithm. Yellow represents the importance score of the first 10 genes screened by the SVM algorithm. Purple represents the importance score of the top 10 genes screened by XGBoost.


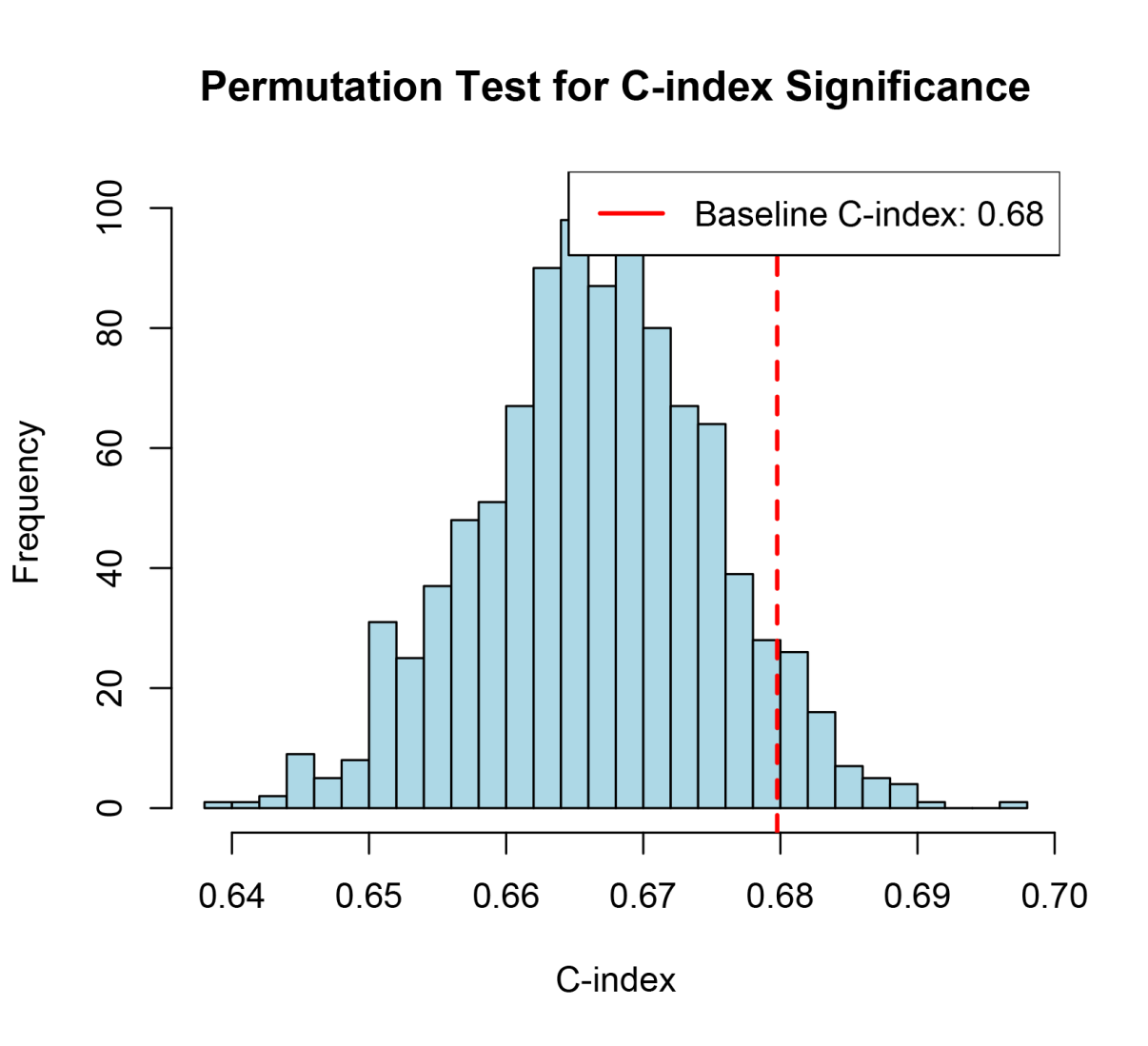


**Supplementary Figure 2.** Histogram of the results of the permutation test. The horizontal axis represents the C-index value, a measure of the model's predictive accuracy. A multiple random C-index distribution was generated by the permutation test to assess whether the predictive power of the model was significantly higher than that of the random model. The vertical axis shows the frequency of occurrence of the C-index values generated by the permutation test in each interval. The histograms illustrate the distribution of C-index values from multiple permutation tests, showing the density of the distribution of C-index in different intervals.


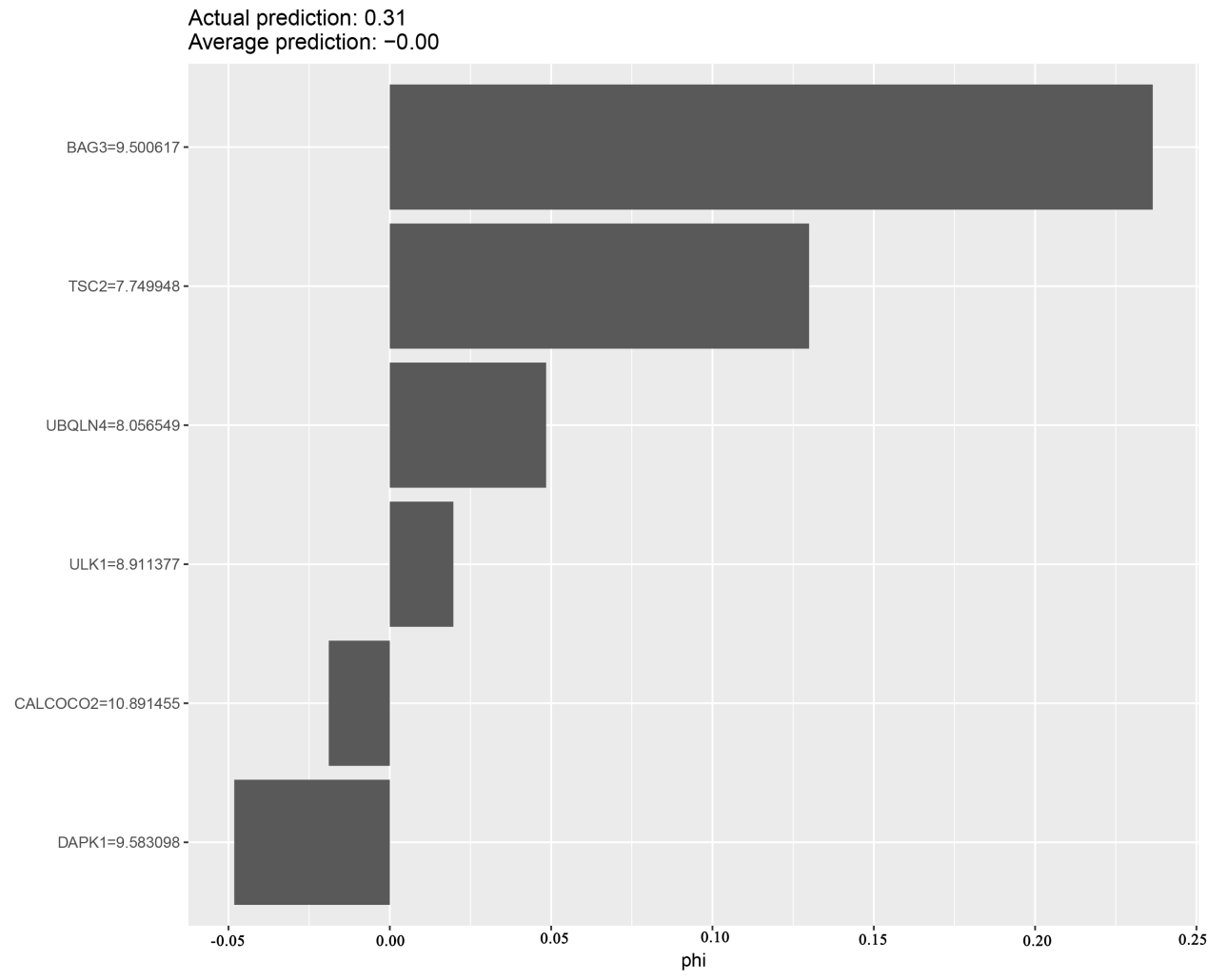


**Supplementary Figure 3.** Interpretation plot of SHAP values for the regression model. The horizontal axis represents the magnitude of the SHAP values.The SHAP values are based on a game-theoretic interpretation method that quantifies the contribution of each gene to the predictions of the Cox regression model. The vertical axis lists the selected genes in the Cox regression model. These are genes that play an important role in the model, selected after stepwise regression and used to explain the predictions of the model.


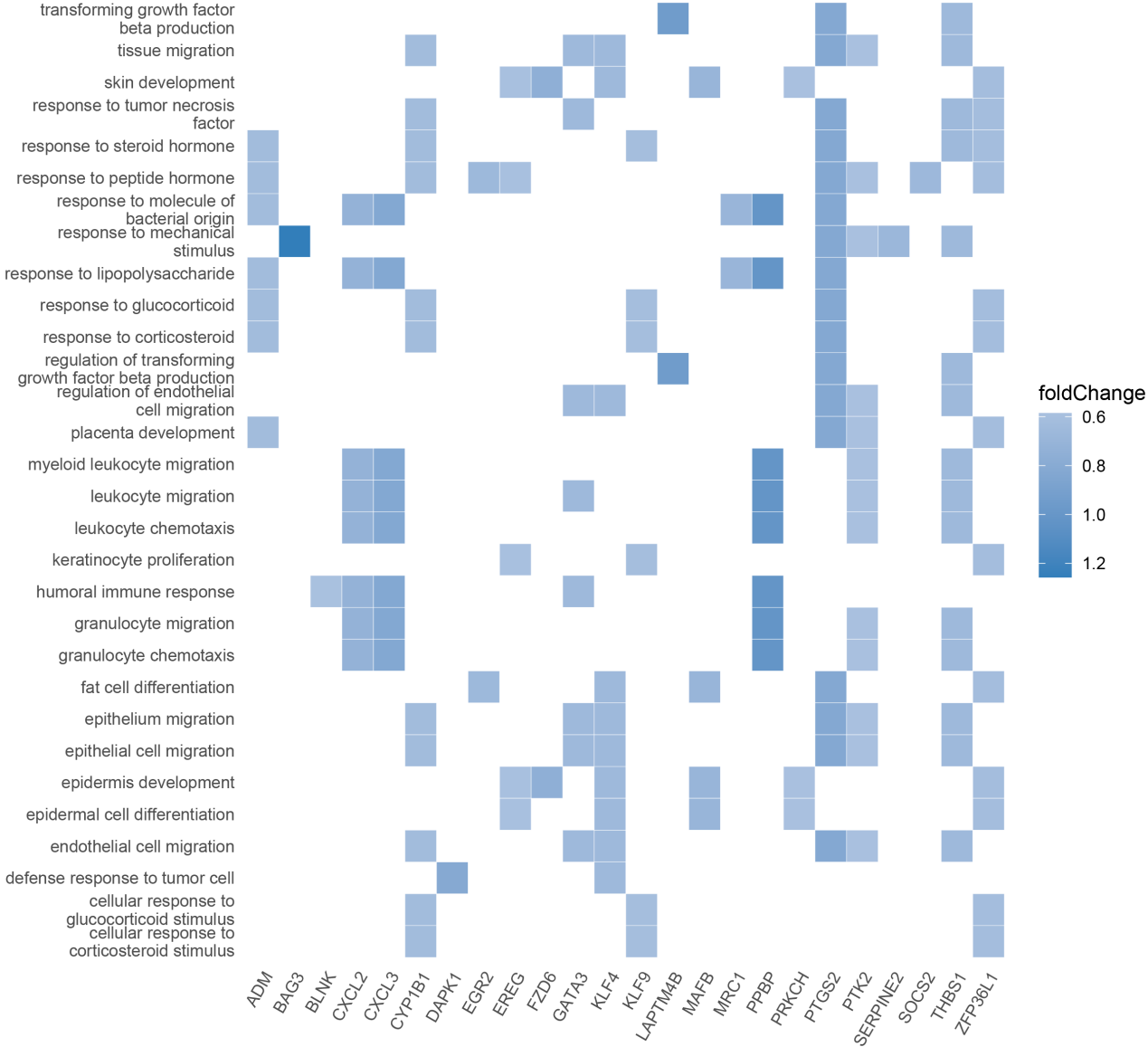


**Supplementary Figure 4.** Heatmap of gene and enrichment term correlations in GO terms. Rows represent GO terms, columns represent genes. Shades of color represent the magnitude of gene expression.


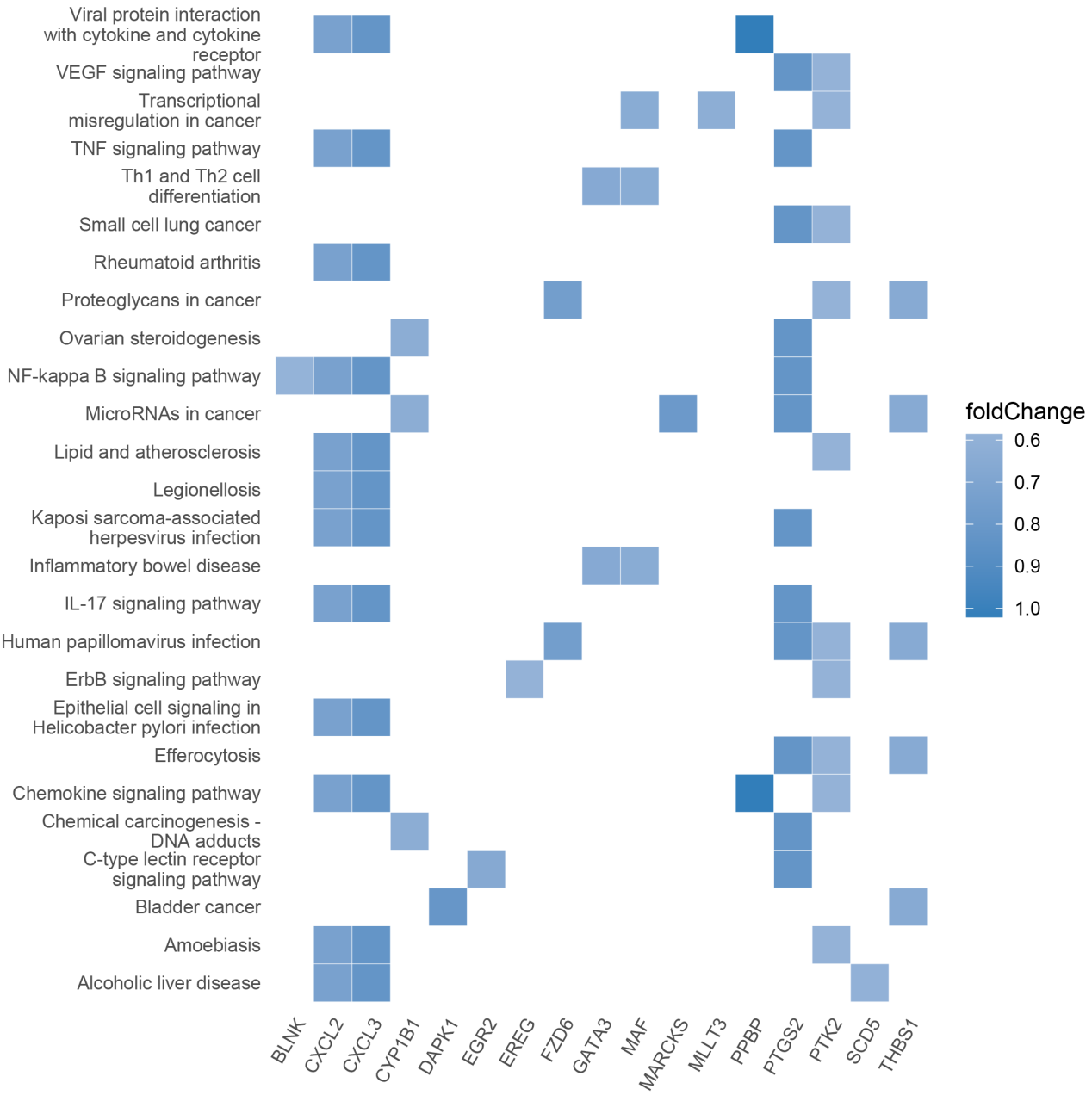


**Supplementary Figure 5.** Heatmap of gene and enrichment pathways correlations in KEGG pathways. Rows represent enrichment pathways, columns represent genes. Shades of color represent the magnitude of gene expression.


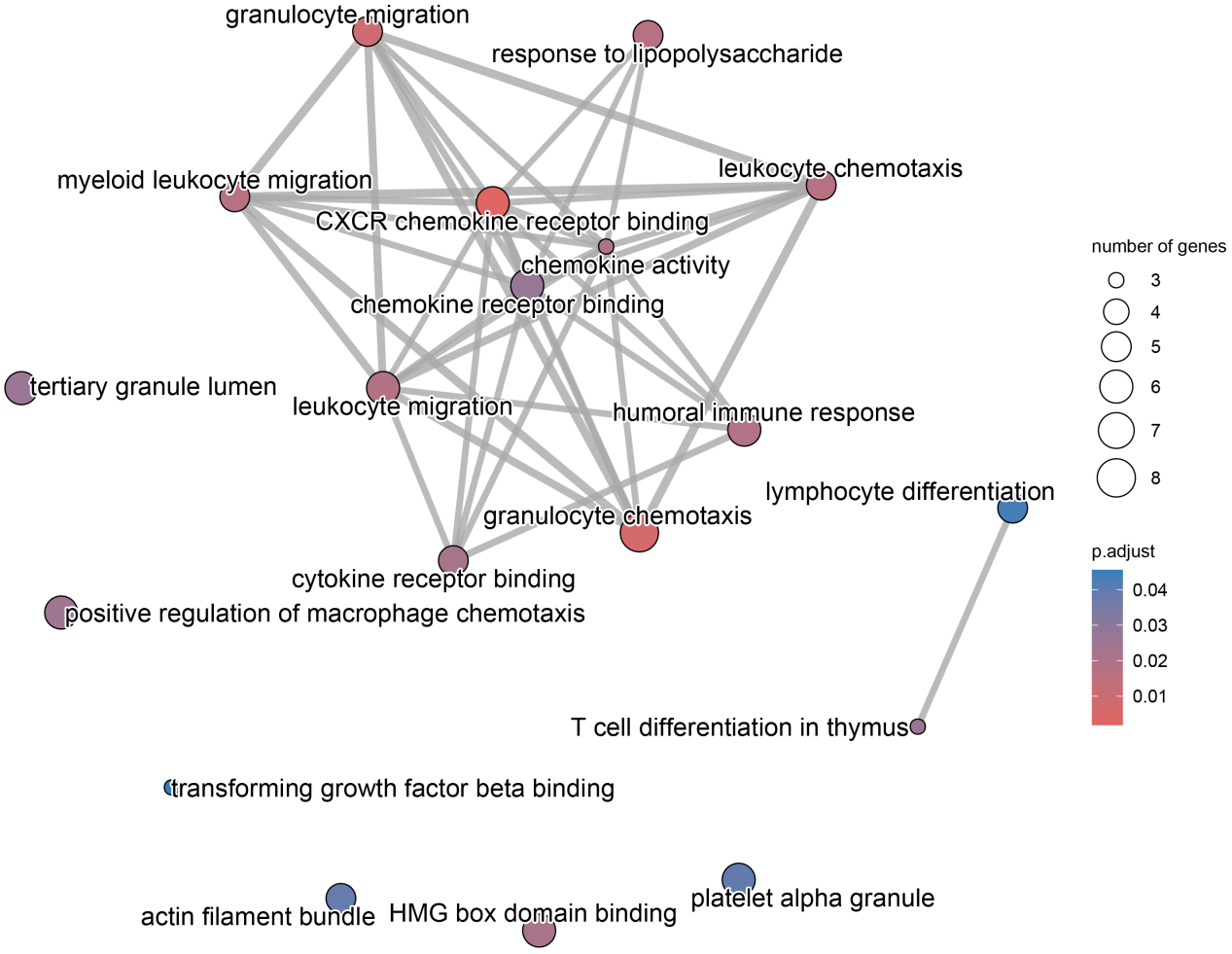


**Supplementary Figure 6.** Network relationships between GO terms. Larger nodes represent more genes enriched in the term. The greater degree of the node represents higher correlation with other terms.


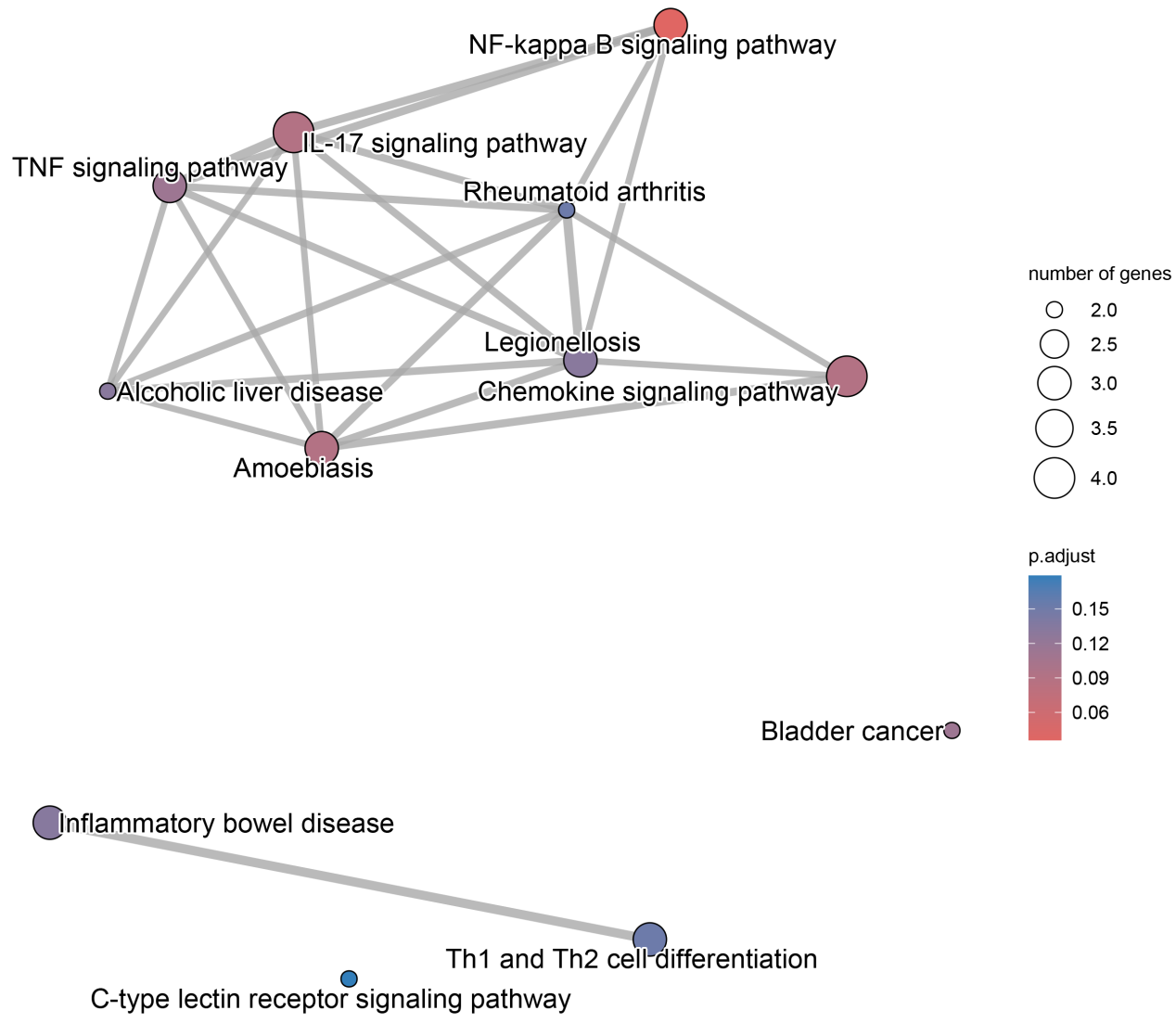


**Supplementary Figure 7.** Network relationships between KEGG-enriched pathways. Larger nodes represent more genes enriched in the pathway. The greater degree of the node represents higher correlation with other pathways.


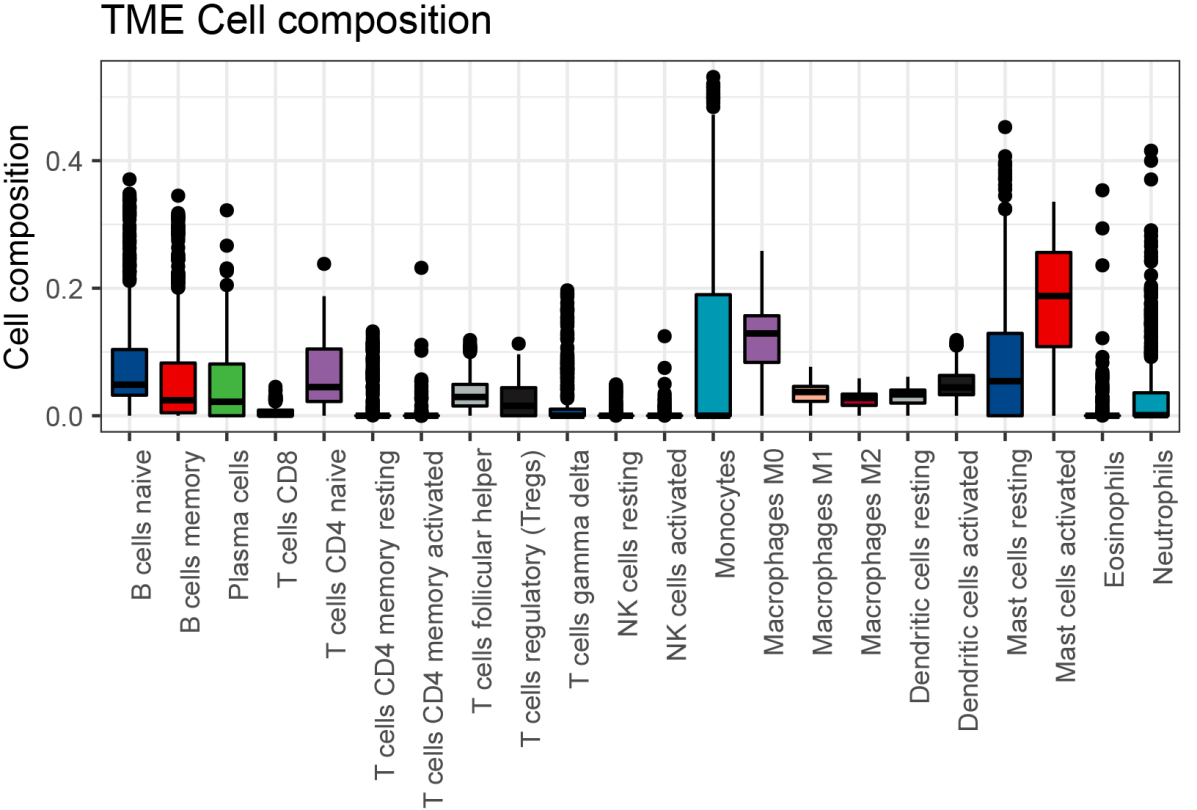


**Supplementary Figure 8.** Box plot of immune cell abundance in leukemia. The horizontal axis represents the proportion of immune cells and the vertical axis represents the types of immune cells.
